# Current global crises and youth mental health in Germany: the role of media use, coping and resources

**DOI:** 10.1007/s00787-026-03092-6

**Published:** 2026-06-13

**Authors:** Anne Kaman, Janine Devine, Michael Erhart, Ann-Kathrin Napp, Franziska Reiss, Christine M. Freitag, Fionna Zoellner, Ulrike Ravens-Sieberer

**Affiliations:** 1 Department of Child and Adolescent Psychiatry, Psychotherapy, and Psychosomatics, University Medical Center Hamburg-Eppendorf, Hamburg, Germany; 2 Alice Salomon University of Applied Science, Berlin, Germany; 3 German Center for Child and Adolescent Health (DZKJ), Partner Site Hamburg, Hamburg, Germany; 4 Department of Child and Adolescent Psychiatry, Psychosomatic Medicine and Psychotherapy, University Hospital Frankfurt, Goethe University, Frankfurt am Main, Germany

**Keywords:** Health-related quality of life, Internalizing symptoms, Future anxiety, Social media exposure, Coping strategies, Psychosocial resources

## Abstract

Young people are currently facing multiple global crises. This study provides an overview of recent data from a population-based German study to examine youth mental health in this multi-crisis context and explores coping strategies and social media use. Data were drawn from eight survey waves (T1/2020–T8/2025) of the nationwide COPSY study, including *n* = 3,312 families and *n* = 2,291 adolescent self-reports. The design combined longitudinal and repeated cross-sectional measurement. Validated instruments assessed health-related quality of life (HRQoL), symptoms of mental disorders, crisis-related worries, social media use, coping strategies, and resources. Data were analyzed descriptively and compared with pre-pandemic reference data. Cross-sectional associations at T8 were examined using chi-square tests and logistic regression adjusted for age and gender. In autumn 2025, the proportions of children and adolescents reporting low HRQoL (22%), mental health problems (23%), anxiety symptoms (25%), or loneliness (18%) remained slightly above pre-pandemic levels. Worries remained widespread, particularly regarding wars (70%), terrorism (62%) and economic problems (57%). Crisis-related future anxiety was cross-sectionally associated with low HRQoL and mental health problems (ORs = 1.8–3.4) in 2025. Social media use was high, with many youths reporting exposure to distressing content. Active coping strategies were used most frequently, while strong personal, familial, and social resources showed potentially protective associations with mental health outcomes (ORs = 0.1–0.2). Despite signs of recovery, elevated crisis-related worries and strong associations between future anxiety and mental health indicate ongoing vulnerability among youth. Strengthening psychosocial resources and promoting safe digital environments deserve greater attention in public health and prevention efforts.

**Supplementary Information:**

The online version contains supplementary material available at https://doi.org/10.1007/s00787-026-03092-6.

## Introduction

Young people currently have to cope with several global crises. In particular, geopolitical events like accelerating climate change with increasing natural disasters, escalating conflicts in Europe and the Middle East, macro-social problems like rising economic insecurity and inflation, and the rapid spread of unregulated social media and artificial intelligence (AI) present substantial challenges for young people. Although the COVID-19 pandemic has ended, pandemic-related experiences and mental health impairments may still shape how children and adolescents perceive and navigate current global challenges.

Recent conceptual work has introduced the notion of a polycrisis to describe situations in which crises across multiple global systems become causally entangled, producing harms greater than the sum of their individual effects [1]. Applied to child and adolescent mental health, this perspective suggests that simultaneous crises such as war, climate change, economic insecurity, and digital transformation may interact and accumulate, thereby intensifying psychological distress beyond isolated exposures. However, this perspective has received only limited empirical attention to date [2–3].

To our knowledge, there is currently no specific theoretical model of youth mental health in times of global crises that conceptualizes how these macro-level stressors interact and influence mental health outcomes. In particular, the potentially mediating and moderating roles of social media exposure, anticipatory stress such as crisis-related future anxiety, coping behaviors, and psychosocial resources remain largely unexplored. We therefore adopt a multi-level framework, viewing current global crises as chronosystemic stressors [4] that affect youth mental health through processes of cumulative risk [5]. Within this framework, both direct and indirect exposure to macro-level crises may manifest in mental health outcomes and future-related anxiety, while coping strategies and psychosocial resources may shape individual vulnerability and resilience.

A recent review [6] summarizes the literature on the psychological and educational impact of local and global crises. In addition, a growing body of individual studies shows that children exposed to disasters, wars, terrorism, or economic hardship – even if exposure is indirect and mediated through news or social media – are at risk of emotional and behavioral problems, stress responses, and worries about their future, i.e. anticipatory stress. Climate-related anxieties have been reported globally, with many young people expressing fears about long-term environmental degradation and a perceived lack of governmental action [7–9]. Likewise, the psychological impact of war and terrorism includes heightened stress, post-traumatic reactions, and disrupted well-being [10–11]. In addition, international studies indicate that financial stress can increase parental distress and family conflict, which in turn may disrupt children’s development and elevate emotional, behavioural, and cognitive difficulties [12–13].

Children and adolescents are particularly vulnerable in the face of these crises because they are still developing cognitively, emotionally, and socially. Limited coping capacities and dependence on adult support make them more susceptible to stress reactions such as anxiety, uncertainty, and irritability [14].

Children’s heightened vulnerability is further shaped by the environments in which they grow up. Over the past decade, children and adolescents in Western societies have been exposed to digital media in ways that were not possible before. Mounting evidence suggests that digital environments can amplify both exposure to crises and emotional responses to them. Increased screen time, problematic social media use, exposure to harmful or distressing content, cyberbullying, and social comparison have all been associated with lower well-being and higher rates of anxiety and depressive symptoms [15]. Although social media can support social connection and emotional expression [16], its conceptual role as a potential mediator of mental health outcomes in times of global instability requires careful examination.

The COVID-19 pandemic marked a turning point in youth mental health worldwide. Meta-analyses and longitudinal studies show substantial declines in health-related quality of life (HRQoL), increased anxiety and depressive symptoms, and heightened stress, loneliness, and behavioral difficulties during the pandemic years [14, 17–20]. While partial recovery has been observed since 2022, many mental health indicators remain worse than before 2020 [21–22]. Recent scientific and policy reports – including the *Lancet Psychiatry Commission on Youth Mental Health* – highlight that these developments constitute a significant challenge for public health and require sustained societal attention [23]. Concerns about climate change, war, terrorism, and economic crises are particularly prevalent and have increased in recent years, even as worries about the COVID-19 pandemic itself have declined [21, 24].

Despite growing public and scientific attention, systematic longitudinal population-based studies that examine the combined and interacting effects of multiple global challenges on youth mental health remain scarce [25]. There is an urgent need for a better understanding of how young people cope with these cumulative challenges and which personal, familial, and social resources might buffer their effects. Given rapidly changing realities and digital transformations in young people’s lives, it is also crucial to understand how social media use shapes risk and resilience within this multi-crisis environment.

The German COPSY (Child Outcomes in PSYchology) study provides a unique opportunity to fill these gaps. Since early 2020, COPSY has followed a population-based sample of children, adolescents, and families across multiple survey waves, using a combined longitudinal and repeated cross-sectional approach and capturing developments throughout the pandemic and beyond. This manuscript presents the main descriptive longitudinal trends across waves as well as cross-sectional analyses from the eighth survey wave (autumn 2025), thus providing an overview of youth mental health in the current post-pandemic period and within the context of intensifying global challenges.

The study aims at:

1. Describing current levels and trajectories in HRQoL, mental health problems, anxiety, depressive symptoms, and loneliness;
2. Assessing current patterns and changes over time in crisis-related worries and future anxieties among children and adolescents;
3. Examining cross-sectional associations between crisis-related future anxiety and mental health outcomes;
4. Describing social media and AI use – including daily duration, distressing content, and online experiences;
5. Describing the use of coping strategies during global crises; and
6. Examining risk and resource factors associated with mental health outcomes.

## Methods

### Study design and sample

The German COPSY study is a population-based longitudinal survey assessing the well-being and mental health of children and adolescents across eight waves from spring 2020 to autumn 2025. The survey waves captured different stages of the COVID-19 pandemic and the subsequent multi-crisis context (T1: 05–06/2020 to T8: 09–10/2025). Pre-pandemic reference data were drawn from the population-based German BELLA study [26] (T0: 2014–2017), which used the same methodology and instruments, allowing direct comparison.

Across the eight COPSY survey waves, between *n* = 1,586 (T1) and *n* = 1,607 (T8) families took part in nationwide online assessments. Families who had participated previously were invited again at each measurement point (longitudinal design), and new families were added as needed to counter age-related attrition and to maintain sociodemographic representativeness across waves (repeated cross-sectional design). Altogether, *n* = 3,312 families with children aged 7 to 23 years contributed data to at least one wave of the study. In addition to parent-reported information, self-report questionnaires were completed by adolescents aged 11 years and older (*n* = 2,291). A detailed participant flow chart showing the number of participants across all survey waves, including longitudinal follow-up and newly recruited families added to compensate for attrition, is provided in Supplementary Figure S1. Although the overall sample includes 7- to 23-year-olds, the present analyses are restricted to participants aged 7 to 21 years, as respondents aged 22 years and older completed an age-adapted questionnaire. Analyses of this older subgroup will be reported separately and are not part of the current paper.

The initial participation rate (T1) was 45.8% of all invited participants at T1, and participants took part in an average of 49.0% of the survey waves (T2–T8). Longitudinal follow-up was enabled through pseudonymized participant identifiers, allowing individual-level linkage across waves while analyses were conducted on de-identified data. To ensure population-level representativeness, data from all waves (T1–T8) were weighted according to the most recent German Microcensus. Weighting was applied at the parent level and included adjustment for children’s school type, age and gender of both parents and children, household size, federal state, family status, and parental employment. This weighting procedure was primarily intended to improve demographic representativeness of each survey wave and does not fully eliminate the possibility of selective attrition bias across repeated participation. Further methodological details are available in Kaman et al. [21].

### Measures

#### Sociodemographic variables

The age and administrative gender of both children and adolescents as well as their parents were recorded. Parents also provided information on their marital status, migration background, and educational attainment. Parental education was classified as low, medium, or high according to the Comparative Analyses of Social Mobility in Industrial Nations (CASMIN) framework [27].

#### Health-related quality of life and mental health

HRQoL was assessed using the self-reported version of the internationally established KIDSCREEN-10 Index [28]. Children and adolescents were categorized as having low, normal, or high HRQoL based on reference data from the national BELLA study (normal HRQoL defined as M_BELLA_+/-1SD_BELLA_). The KIDSCREEN-10 showed good internal consistency in the current sample (Cronbach’s α = 0.86).

The parent-reported Strengths and Difficulties Questionnaire (SDQ) [29] was used to assess mental health problems in children from age seven onwards. A total difficulties score was calculated across 20 items, with higher scores indicating more severe mental health problems. To classify children and adolescents *with* and *without* mental health problems (i.e., *abnormal/borderline* vs. *normal*), established cut-offs were applied [30]. Internal consistency of the SDQ was good in the present sample (Cronbach’s α = 0.85).

Children and adolescents self-reported anxiety via the General Anxiety subscale of the Screen for Child Anxiety Related Disorders (SCARED) [31]. A total score was derived from the nine items, with higher values indicating more severe symptoms. Based on established cut-offs, participants were grouped into those *with* and *without* anxiety [31]. The SCARED showed excellent internal consistency in the present sample (Cronbach’s α = 0.90).

Depressive symptoms were self-reported using seven items of the German version of the Center for Epidemiological Studies Depression Scale (CES-DC) [32], which measures impairment due to depressive symptoms over the past week. A sum score was calculated, with higher scores reflecting more symptoms. Participants were categorized into groups *with* and *without* noticeable depressive symptoms using established cut-offs [33]. The CES-DC demonstrated good internal consistency in the current sample (Cronbach’s α = 0.87).

Loneliness during the past week was assessed with a single item from the KIDSCREEN-10 (“Have you felt lonely?”) [28], rated on a 5-point Likert scale (*never*,* almost never*,* sometimes*,* almost always*,* always*). Following prior publications and for comparability across waves, responses of *sometimes*, *almost always* or *always* were classified as indicating loneliness [22]. For all longitudinal trend analyses across waves (T1–T8), this single-item measure was used consistently. In addition, at T8 loneliness was also assessed using the 4-item version of the UCLA Loneliness Scale [34] to provide a more comprehensive assessment in the most recent wave. Higher scores indicate greater loneliness over the past 12 months. A cut-off similar to previous research [22] was applied. The UCLA measure was used exclusively for cross-sectional regression analyses at T8. In the present sample, the UCLA demonstrated good internal consistency (Cronbach’s α = 0.87).

#### Crisis-related worries and crisis-related future anxiety

From T5 onwards, children’s and adolescents’ worries about current crises were assessed using single-item questions (e.g., “How worried are you about the following issues at the moment?”: pandemic, wars, economic crisis, climate crisis, terrorism/attacks). At T7, the list of items was expanded to include worries about societal division and migration. All items were rated on a 5-point Likert scale ranging from *not at all* to *very worried*.

Crisis-related future anxiety was assessed from T6 onwards using an adapted version of the Dark Future Scale for Children (DFS-K) [35–36]. The adapted version was designed to capture crisis-specific future concerns in the context of ongoing global challenges. The 4-item scale measures anxiety about the future in relation to current crises, including worsening life conditions, family finances, and limitations on hobbies and personal goals. Adolescents rated their agreement on a scale from 0 (*no*,* never / does not apply*) to 3 (*yes*,* often / fully applies*). Higher scores indicate greater crisis-related future anxiety. In the present sample, the scale showed excellent internal consistency (Cronbach’s α = 0.92).

#### Use of social media and AI

Daily duration of social media use was assessed with a single self-report item with eight response categories ranging from “*never*” to “*more than 7 hours per day / almost all day*”. Children and adolescents who reported using social media were additionally asked about social media–related burden using two items assessing (1) exposure to distressing content and (2) experiences of exclusion or devaluation. Response options ranged from 1 (*strongly disagree*) to 5 (*strongly agree*). For descriptive purposes, both broader endorsement (responses 3–5, indicating at least partial agreement) and stronger burden (responses 4–5, indicating stronger agreement) were reported. At T8, the use of artificial intelligence (AI) was assessed with a dichotomous item (*yes/no*; examples provided such as ChatGPT, Snapchat MyAI, Google Gemini). Adolescents who indicated using AI were subsequently asked about their purposes of use across seven categories (school/homework, fun, curiosity, information seeking, creating images or videos, communication, and discussing personal worries), with multiple responses permitted.

#### Coping strategies

Coping strategies were assessed using the German self-report version of the KIDCOPE for adolescents [37–38]. The KIDCOPE captures a broad range of cognitive and behavioral coping responses. The instrument was pre-tested for comprehensibility with German children aged 11 years and older, and minor wording adjustments were made accordingly. The KIDCOPE consists of 11 items assessing ten distinct coping strategies that can be grouped into three domains: active (problem solving, emotional regulation, social support, cognitive restructuring), avoidant (distraction, social withdrawal, wishful thinking, resignation), and negative coping (self-criticism, blaming others) [39]. Respondents rated the frequency of using each strategy on a 4-point Likert scale (0–3; *not at all* to *almost all the time*), in response to the instruction to indicate how often they use each strategy when feeling stressed or worried. If a strategy was reported as being used, its perceived efficacy (“How much did it help?”) was assessed on a scale from 0 (*not at all*) to 4 (*very much*).

#### Risk and resource factors

Information on parental educational level, migration background, and living space was recorded. Children and adolescents were assigned to a risk group if their parents had a low level of education and either a migration background or lived in close quarters (< 20 m² living space per household member). In addition, children and adolescents were classified as part of the risk group if their parents reported mental health problems (e.g., depressive symptoms, diagnosed mental illness, or severe concerns related to current crises). Parental depressive symptoms were assessed using the Patient Health Questionnaire (PHQ-8) [40]. Based on 8 items, a total score between 0 and 24 was calculated. This composite risk group indicator was designed to capture cumulative psychosocial burden by combining structural and familial risk factors, in line with previous COPSY studies. Its operationalization was based on both theoretical considerations and empirical evidence from previous analyses of psychosocial risk factors within the COPSY study.

Personal resources such as problem-solving skills and optimism were assessed using the Personal Resources Scale [41]. Perceived social support was measured with four items from the Social Support Scale [42]. To assess family cohesion, four items from the “Family Cohesion” subscale of the Family Climate Scale were used [43]. For all three scales, items referred to experiences during the past week, and sum scores were calculated and transformed into values between 0 and 100, with higher values indicating stronger resources. Children and adolescents were classified as having pronounced resources if they reported above-average scores on each of the three scales compared to reference data from the BELLA study. The three scales showed good internal consistency, with Cronbach’s alpha values of α = 0.82 for personal resources, α = 0.88 for social support, and α = 0.88 for family cohesion.

### Statistical analyses

To assess how HRQoL and mental health changed during times of global crises, the proportions of children and adolescents with low HRQoL, noticeable mental health problems, depressive symptoms, anxiety symptoms, and loneliness (KIDSCREEN item) were calculated for each survey wave (T1–T8) and compared with pre-pandemic estimates from the BELLA study (2014–2017). Statistically significant differences were tested using chi-square analyses, and effect sizes were quantified with Cramer’s V. To quantify statistical uncertainty, standard errors for all HRQoL and mental health parameters are reported in the main manuscript. Full 95% confidence intervals are presented in a separate supplementary table. No Bonferroni correction was applied across comparisons over waves and outcomes, as these analyses were primarily descriptive, and the tests were not statistically independent due to repeated measurements and correlated outcomes.

Crisis-specific worries (pandemic, wars, economic crisis, climate crisis, terrorism/attacks) at T8 (2025) were first analyzed descriptively and then compared with data from previous survey waves (T5–T7). In addition, the four items of the Dark Future Scale were examined using descriptive statistics. Logistic regression models were then used to analyze the association between crisis-related future anxiety (independent variable) and HRQoL, mental health problems, depressive symptoms, anxiety symptoms, and loneliness assessed with the UCLA (dependent variables) at T8, controlling for age, gender, and their interaction terms.

Information on social media use, experiences in social media, and the use of AI was analyzed descriptively. Coping strategies were analyzed descriptively. Multiple logistic regression analyses were used to investigate whether belonging to the risk group is associated with increased or decreased odds (OR) of low HRQoL, mental health problems, anxiety symptoms, depressive symptoms, and loneliness (UCLA) at T8. Age, gender, and their interaction terms were included as covariates. In an extended model, the presence of adequate personal, familial, and social resources was additionally integrated into the regression models as a further predictor.

No imputation of missing data was performed. Cases with missing values on any of the variables included in a particular analysis were excluded from that analysis (complete-case approach).

## Results

### Sociodemographics

In total, *n* = 3,312 families with children and adolescents aged 7 to 21 years (M = 14.8; SD = 4.6; 49.3% female) participated in at least one of the eight COPSY survey waves (T1–T8). Of these, *n* = 2,291 adolescents aged 11 to 21 years (M = 17.1; SD = 3.5; 50.3% female) completed the self-report questionnaires. The majority of parents had a medium level of education. Approximately one fifth of the children and adolescents had a migration background, and one fifth of parents were single parents. Additional sociodemographic information is presented in Table 1.

**Table 1:** Sociodemographic characteristics of the COPSY sample

	Children and adolescents (parent reports) (*n* = 3,312)		Children and adolescents (self-reports) (*n* = 2,291)	
	*n* (%)	M (SD)	*n* (%)	M (SD)
Age		14.8 (4.6)		17.1 (3.5)
7–10 years	756 (22.8)			
11–13 years	656 (19.8)		444 (19.4)	
14–17 years	815 (24.6)		765 (33.4)	
18–21 years	1085 (32.8)		1082 (47.2)	
Gender				
Male	1,661 (50.1)		1,123 (49.1)	
Female	1,634 (49.3)		1,152 (50.3)	
Other	15 (0.5)		14 (0.6)	
Age of the parent		46.2 (7.9)		48.5 (7.4)
Gender of the parent				
Male	1,485 (44.8)		996 (43.5)	
Female	1,824 (55.1)		1,293 (56.4)	
Other	3 (0.1)		2 (0.1)	
Parental education				
Low	480 (14.6)		351 (15.4)	
Medium	1,961 (59.6)		1,344 (59.0)	
High	849 (25.8)		584 (25.6)	
Migration background				
No	2,704 (82.1)		1,871 (82.3)	
Yes	589 (17.9)		402 (17.7)	
Single parent				
No	2,731 (82.9)		1,851 (81.4)	
Yes	562 (17.1)		422 (18.6)	

#### Health-related quality of life and mental health in children and adolescents

**Fig. 1:**
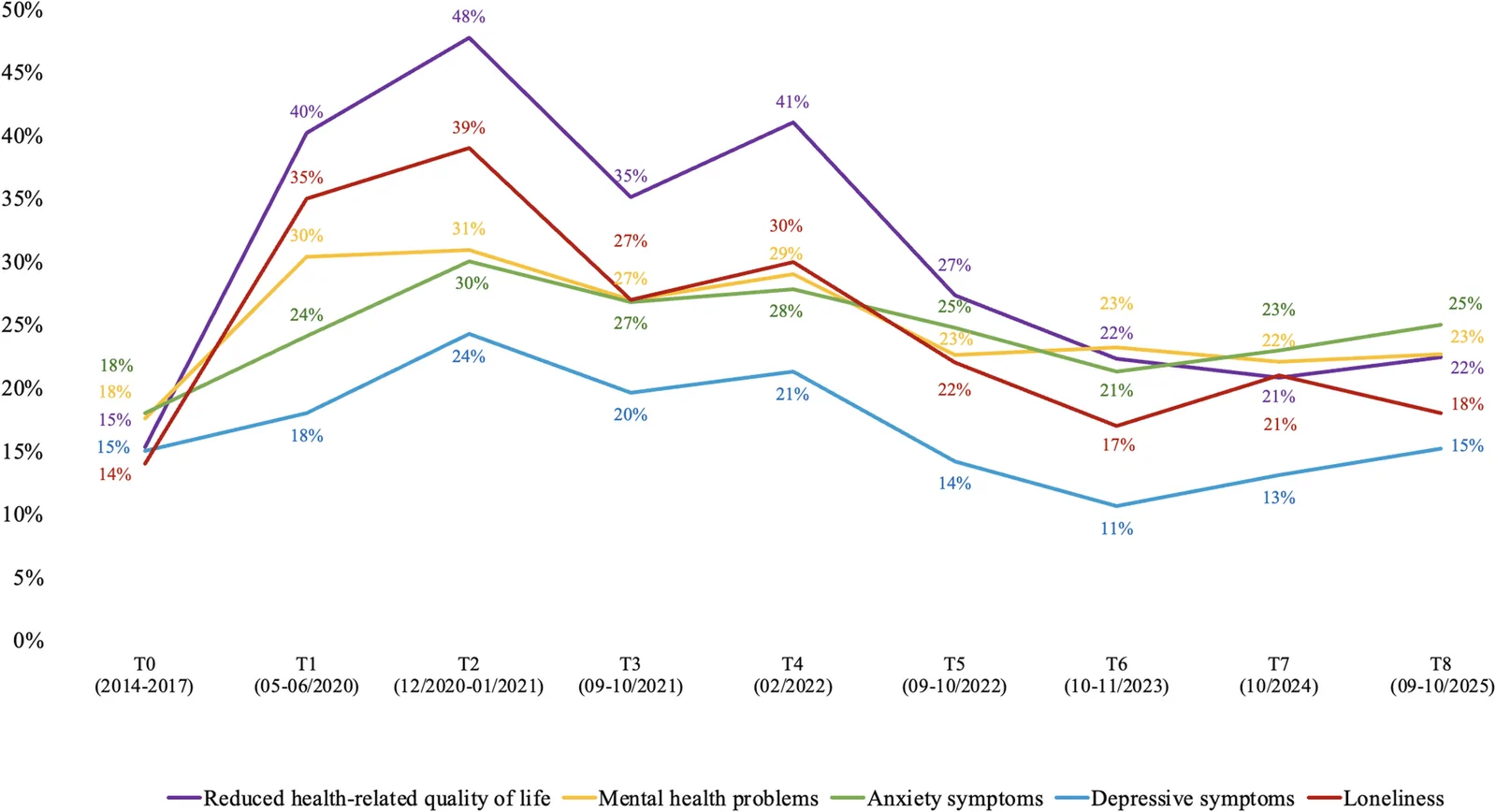
Longitudinal trends in the proportion of children and adolescents with low HRQoL, mental health problems, anxiety symptoms, depressive symptoms and loneliness. Results from the BELLA study (2014–2017) and the COPSY study (2020–2025). Note. Statistically significant changes were observed between T0 and T1 for HRQoL (*p* < 0.001, V = 0.28), mental health problems (*p* < 0.001, V = 0.15), anxiety symptoms (*p* < 0.001, V = 0.08), depressive symptoms (*p* = 0.075, V = 0.04), and loneliness (*p* < 0.001, V = 0.24); between T1 and T2 for HRQoL (*p* < 0.001, V = 0.08), anxiety symptoms (*p* = 0.002, V = 0.08), and depressive symptoms (*p* < 0.001, V = 0.08); between T2 and T3 for HRQoL (*p* < 0.001, V = 0.13), mental health problems (*p* = 0.013, V = 0.04), depressive symptoms (*p* = 0.007, V = 0.06), and loneliness (*p* < 0.001, V = 0.12); between T3 and T4 for HRQoL (*p* = 0.004, V = 0.06); between T4 and T5 for HRQoL (*p* < 0.001, V = 0.14), mental health problems (*p* < 0.001, V = 0.07), depressive symptoms (*p* < 0.001, V = 0.09), and loneliness (*p* < 0.001, V = 0.09); between T5 and T6 for HRQoL (*p* = 0.005, V = 0.06), anxiety symptoms (*p* = 0.047, V = 0.04), depressive symptoms (*p* = 0.010, V = 0.05), and loneliness (*p* = 0.002, V = 0.06); and between T6 and T7 for loneliness (*p* < 0.044, V = 0.04). No statistically significant changes were observed between T7 to T8. Between T0 and T8, statistically significant changes were observed for HRQoL (*p* < 0.001, V = 0.09), mental health problems (*p* < 0.001, V = 0.06) and anxiety symptoms (*p* < 0.001, V = 0.09). Standard errors for these outcomes varied across the waves, ranging from 1.17 to 1.54 for low HRQoL, 0.97 to 1.16 for mental health problems, 1.19 to 1.40 for anxiety symptoms, 0.89 to 1.31 for depressive symptoms, and 1.09 to 1.49 for loneliness

The HRQoL and mental health of children and adolescents deteriorated markedly at the beginning of the pandemic compared with pre-pandemic data (2014–2017) from the BELLA study. Figure 1 presents the proportions of children and adolescents who showed low HRQoL, mental health problems, elevated anxiety, depressive symptoms, or loneliness. The highest prevalence rates were observed during the winter of 2020/21 (T2): nearly half (48%) reported low HRQoL, 39% experienced loneliness, approximately one third reported mental health problems (31%) and anxiety symptoms (30%), and one quarter (24%) reported depressive symptoms. In 2022 and 2023, HRQoL and mental health indicators improved. However, this positive trend did not continue into 2024 (T7) and 2025 (T8). In 2025, 22% of children and adolescents still reported low HRQoL, 23% showed mental health problems, 25% reported anxiety symptoms, and 18% reported loneliness. These prevalence rates remain approximately 5% points above the pre-pandemic levels observed in the BELLA study. Only depressive symptoms recovered to pre-pandemic values; however, since autumn 2024 (T7), a renewed (non-significant) upward trend has been observed (see Fig. 1). An overview of standard errors for each outcome is provided in the notes of Fig. 1. The corresponding 95% confidence intervals for all estimates can be found in the electronic Supplement (Supplementary Table S1).

#### Crisis-related worries and future anxiety

**Fig. 2:**
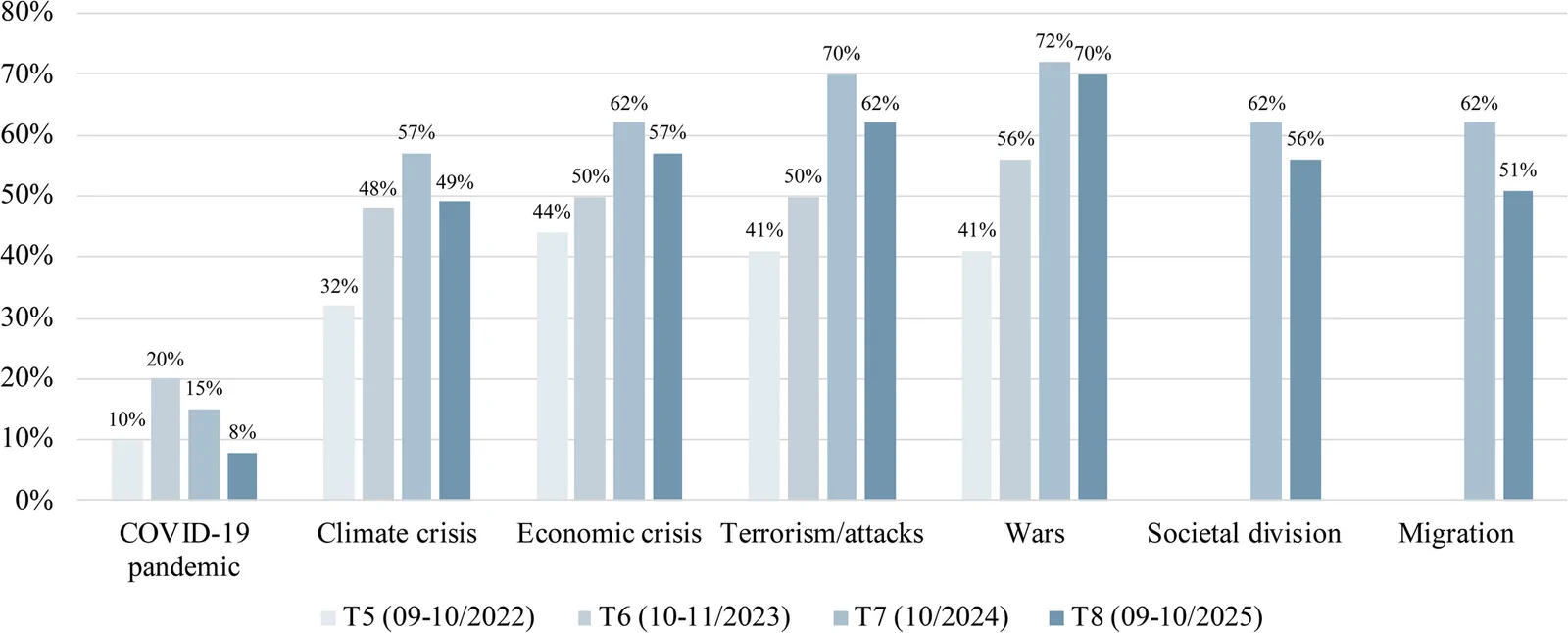
Proportion of children and adolescents who report being moderately to very worried about current crises (2022–2025)

Over the past three years, crisis-related worries among children and adolescents increased significantly from 2022 to 2024 (*p* < 0.001; V = 0.11–0.28), with wars and terrorism/attacks emerging as their most prominent concerns in 2024 (see Fig. 2). From T7 to T8 (2024–2025), worries about wars remained stable at a high level (70%; no significant change), while worries about other crises showed small but significant declines. Specifically, worries related to the pandemic (*p* < 0.001; V = 0.11), the economic crisis (*p* = 0.032; V = 0.05), the climate crisis (*p* < 0.001; V = 0.08), terrorism/attacks (*p* < 0.001; V = 0.09), societal division (*p* = 0.008; V = 0.06), and migration (*p* < 0.001; V = 0.11) slightly decreased. However, substantial proportions of young people still reported being *moderately*, *quite* or *very* worried about terrorism/attacks (62%), the economic crisis (57%), societal division (56%), migration (51%), and the climate crisis (49%) in 2025.

Data on crisis-related future anxiety show that in autumn 2025 (T8), 59% of children and adolescents *sometimes* or *often* fear that the current crises will persist for a long time. Moreover, 52% worry that their living conditions will deteriorate, and 51% fear that their family will soon be able to afford less. In addition, 30% of young people are concerned that they may no longer be able to finish school or pursue their desired career.

#### Associations between crisis-related future anxiety and mental health

Children and adolescents who reported crisis-related future anxiety in autumn 2025 (T8) were more likely to also report low HRQoL, mental health problems, anxiety, depressive symptoms, and loneliness at the same time. Logistic regression analyses showed that a one-point increase in the mean score of crisis-related future anxiety was associated with 1.8 times higher odds of mental health problems in 2025. Slightly stronger associations were observed for low HRQoL (OR = 2.1), depressive symptoms (OR = 2.4), loneliness (OR = 3.0), and anxiety symptoms (OR = 3.4) (see Table 2). The reported odds ratios indicate meaningful associations between crisis-related future anxiety and mental health outcomes. However, the relatively low pseudo-R² values of the logistic regression models (0.06 to 0.19) suggest only modest explanatory power.

**Table 2:** Cross-sectional associations between crisis-related future anxiety and mental health among children and adolescents in autumn 2025

	Health-related quality of life (KIDSCREEN)		Mental health problems (SDQ)		Depressive Symptoms (CES-DC)		Anxiety Symptoms (SCARED)		Loneliness (UCLA)	
	OR	95% CI	OR	95% CI	OR	95% CI	OR	95% CI	OR	95% CI
Gender (Ref. Male)										
Female	0.851	0.47–1.53	0.874	0.45–1.71	**2.171**	**1.05–4.51**	**2.223**	**1.24–3.97**	1.291	0.51–3.27
Age groups (Ref. 11–13 years)										
14–17 years	1.098	0.67–1.80	1.390	0.80–2.41	**1.985**	**1.02–3.86**	1.336	0.78–2.28	1.594	0.72–3.51
18–21 years	1.200	0.70–2.05	0.595	0.30–1.20	**2.415**	**1.20–4.85**	1.129	0.62–2.04	1.186	0.48–2.90
Gender * Age group										
Female * 14–17 years	1.147	0.55–2.42	0.444	0.18–1.10	0.519	0.21–1.27	0.647	0.31–1.36	1.092	0.36–3.31
Female * 18–21 years	1.220	0.56–2.65	1.002	0.36–2.77	**0.358**	**0.14–0.91**	0.782	0.36–1.72	0.400	0.11–1.51
Crisis-related future anxiety	**2.136**	**1.73–2.63**	**1.758**	**1.37–2.26**	**2.411**	**1.89–3.08**	**3.364**	**2.68–4.22**	**2.974**	**2.16–4.09**
Hosmer-Lemeshow GoF	*p* = 0.002		*p* = 0.262		*p* < 0.001		*p* = 0.533		*p* = 0.364	
Nagelkerke R-square	0.082		0.062		0.101		0.190		0.123	

Multiple logistic regression analyses examining associations between crisis-related future anxiety and mental health among children and adolescents, controlling for age and gender groups. *OR*  Odds Ratio, *CI*  Confidence Interval, *GoF*  Goodness of Fit; bold values indicate statistically significant results

#### Use of social media and AI

**Fig. 3:**
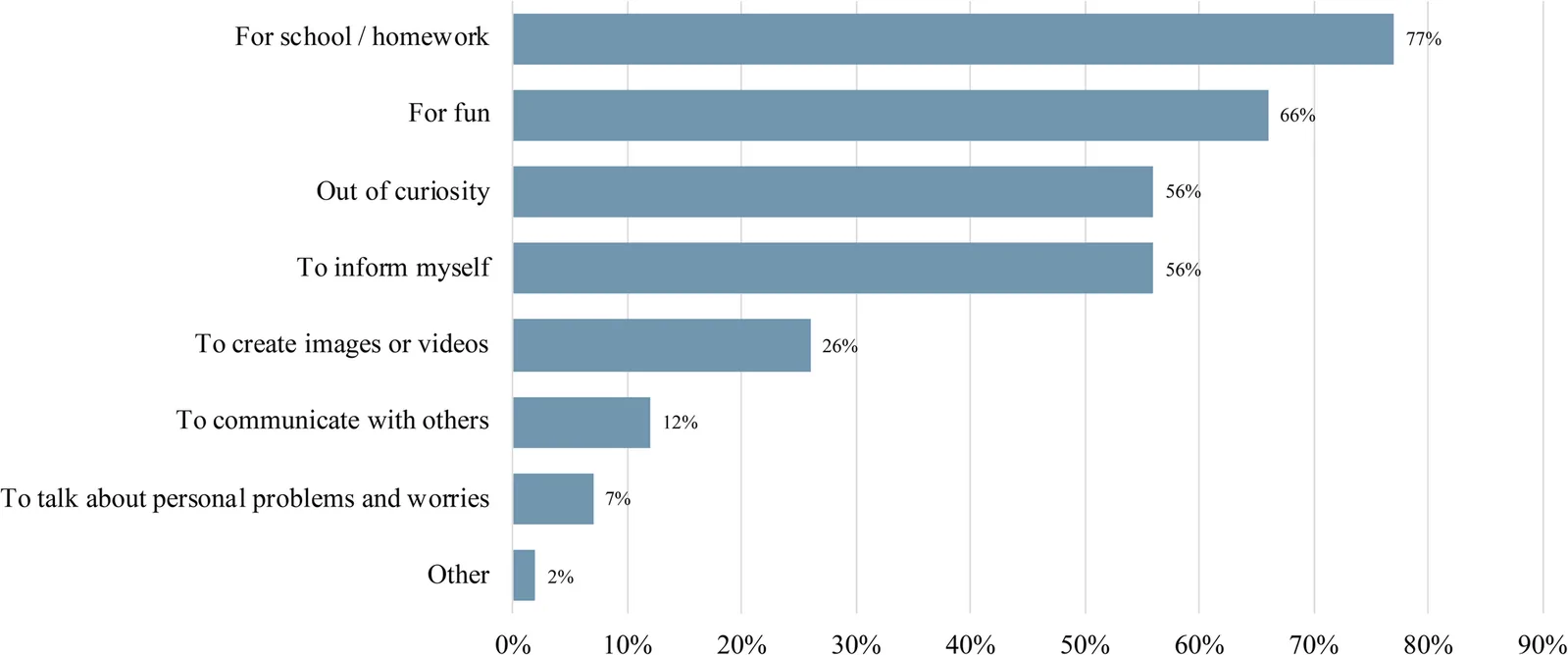
Purposes of artificial intelligence use among children and adolescents (2025)

In autumn 2025 (T8), 21% of children and adolescents reported using social media for at least four hours per day. In addition, 39% of adolescents reported at least partial exposure to distressing content on social media, whereas 9% indicated stronger agreement with this experience. Similarly, 22% reported at least some burden due to exclusion or devaluation in online environments, while 8% reported more pronounced burden. Almost two thirds of youth (63%) reported using AI. Among AI users, the majority (77%) used AI for school or homework, and two thirds (66%) used it for fun. More than half reported using AI out of curiosity (56%) or to obtain information (56%). Around a quarter used AI to create images or videos, whereas only a small proportion used AI to communicate with others or to talk about personal problems and worries (see Fig. 3).

#### Coping strategies used to manage global crises

**Fig. 4:**
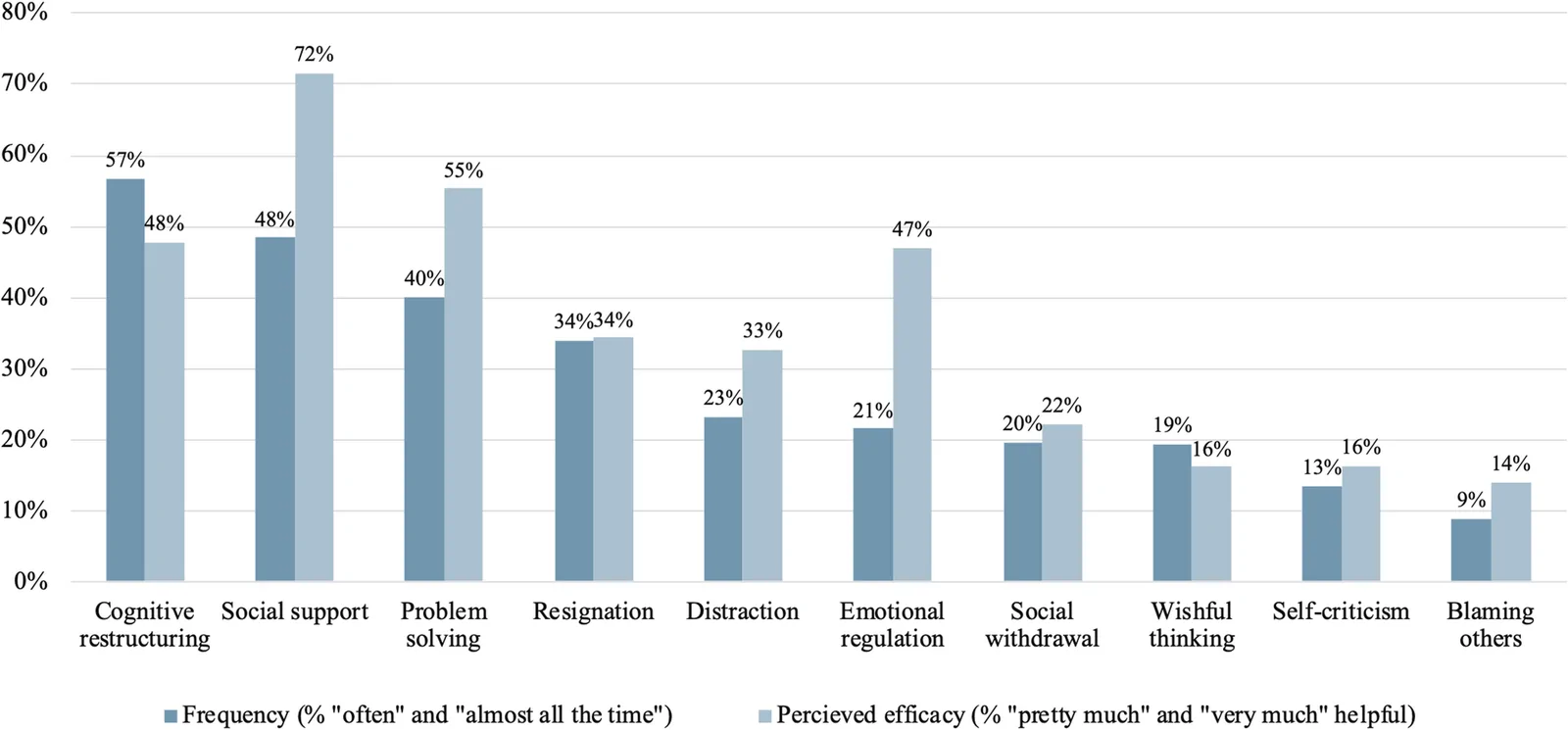
Use and perceived efficacy of coping strategies among children and adolescents (2025)

Children and adolescents reported using a wide range of strategies to cope with current global crises in 2025 (T8) (see Fig. 4). Overall, active coping strategies were most frequently endorsed. More than half of the respondents (57%) reported focusing on positive aspects of the situation (cognitive restructuring), and 48% indicated that they seek support within their families or friends. Around 40% reported engaging in problem-solving to address challenges arising from the crises. At the same time, avoidant strategies were also commonly used. About one third (34%) of the youth reported resignation, and approximately one quarter (23%) used distraction, such as watching television, engaging in screen-based activities, or turning to other leisure activities. Negative coping strategies, such as self-blame or blaming others, were reported least frequently, with 9% to 13% of respondents endorsing them. When asked about the perceived efficacy of these coping strategies, young people rated active coping as the most effective (see Fig. 4). Social support was considered the most helpful strategy, with 72% reporting that it helped them *quite a lot* or *very much*. This was followed by problem-solving (55%), cognitive restructuring (48%), and emotional expression – such as calming down, relaxing, praying, or going for a walk (47%).

#### Risk and resource factors

Around 17% of children and adolescents were particularly vulnerable in terms of their mental health and belonged to the predefined risk group. Risk factors included low parental education, parental mental health problems, a migration background, and limited living space. These children and adolescents had a 1.5 times higher risk (OR) of reduced HRQoL and a 2.0 times higher risk of mental health problems in autumn 2025. In contrast, 52% were classified as having pronounced personal, familial, and social resources. These children and adolescents had a substantially reduced risk of poor mental health outcomes. Specifically, higher resource levels were associated with a five- to tenfold lower likelihood of low HRQoL (OR = 0.1), mental health problems (OR = 0.1), anxiety symptoms (OR = 0.1), and depressive symptoms (OR = 0.2). Detailed results of the full logistic regression models are provided in Supplementary Table S2. To examine the robustness of these findings, sensitivity analyses were conducted using a continuous combined index of the three resource scores (range: 0–100). These analyses yielded comparable results, with odds ratios ranging from 0.88 to 0.94, confirming the overall pattern of the main analyses.

## Discussion

This study provides updated evidence on how children and adolescents in Germany are dealing with the psychological consequences of multiple global crises in autumn 2025. While some improvements in well-being were observed over the past years, the findings indicate that the mental health of young people has not completely returned to pre-pandemic levels as reflected by the BELLA reference period (2014–2017). Instead, a considerable proportion of children and adolescents continues to experience reduced HRQoL, mental health problems, increased rates of anxiety and depressive symptoms, and loneliness. Moreover, crisis-related worries and future anxiety remain widespread, suggesting that today’s youth are confronted with a persistently challenging social and global environment. Active coping and strong psychosocial resources can be seen as key protective factors, whereas high social media use, exposure to distressing content, and widespread AI use became apparent as relevant features of adolescents’ current digital environments that may reflect additional psychosocial challenges.

Consistent with findings from earlier COPSY survey waves [21] and other international post-pandemic studies [44–45], HRQoL and mental health indicators have improved since the peak of the COVID-19 pandemic but remain elevated compared to pre-pandemic levels. In 2025, around one in four children and adolescents report poor HRQoL or mental health problems, and one in five experience loneliness. These findings indicate that the emotional consequences of the pandemic and subsequent crises may be long-lasting, although broader secular developments in adolescent mental health and digital environments should also be considered. However, the majority of children and adolescents report levels of well-being within the normal range, indicating substantial adaptive capacities and resilience at the population level.

Crisis-related worries present a further area of concern. Wars and terrorism/attacks are still the most prominent concerns, with the proportion of worried youth remaining stable at a high level. Although worries about other crises – such as economic instability, climate change, societal division, migration, and terrorism – decreased slightly between 2024 and 2025, these reductions were small in magnitude. Nevertheless, the continuing prevalence of crisis-related worries underscores that young people are coping with an accumulation of global stressors that shape their daily experiences and future expectations. These findings are consistent with recent international studies [44] as well as German studies [24, 46–47] indicating that crisis-related worries and future fears are widespread among young people. Together, these findings support the conclusion that worries about global crises are deeply embedded in the everyday lives of today’s youth and should draw greater attention in mental health research and policy.

The cross-sectional associations between crisis-related future anxiety and all examined mental health outcomes further underline the close link between perceived global uncertainty and youth psychological well-being. Higher levels of future anxiety were correlated with substantially increased odds of low HRQoL, mental health problems, depressive symptoms, anxiety, and loneliness. While these cross-sectional associations do not allow for causal inference, the pattern mirrors emerging international evidence showing that future-related anxieties – including those tied to climate change, economic uncertainty, or geopolitical threats – tend to co-occur with internalizing symptoms in youth [8]. This is also consistent with a recent review by Jibai et al. [6], which summarises how various global crises are associated with lasting psychological strain among children and adolescents. At the same time, reciprocal processes are plausible: adolescents with existing mental health problems may be more prone to negative appraisals of global crises, stronger future-related fears, or greater exposure to distressing content and burdensome online experiences. Further, crisis-related future anxiety represents one important correlate among several factors associated with youth mental health and should not be interpreted as an exclusive explanatory mechanism. In line with earlier COPSY findings, the present results reinforce that crisis-related future anxiety represents an important correlate of mental health in the current multi-crisis context.

Patterns of social media use and online experiences further illustrate the complexity of the psychosocial landscape in which young people are growing up. A considerable proportion of children and adolescents use social media for several hours per day, frequently encounter distressing content, and report experiences of exclusion or devaluation online. While many young people use AI primarily for information and school-related purposes, a minority turn to digital tools for emotional support. These findings indicate both opportunities and risks associated with the use of digital media. Recently, international evidence has been accumulating on the dual role of social media in adolescent development [15]. High and problematic social media use has repeatedly been linked to increased symptoms of anxiety and depression in adolescents [16, 48], and may act as a moderator between external crises and mental health outcomes, which needs to be further studied. However, until more scientific knowledge on the benefits and harms of social media exists, we would like to underscore the need for careful monitoring of young people’s digital engagement and its potential mental health impacts. In addition, recent national expert reports highlight the need for clearer frameworks to ensure safe digital environments for minors, particularly regarding exposure to harmful content and algorithmically amplified risks. While strengthening digital literacy among families and schools remains essential, some experts argue that effective protection cannot rely on individual actions alone. Rather, platform-level design safeguards, transparent information about digital risks, and age-appropriate default settings are increasingly seen as necessary components of a comprehensive youth mental health strategy in the digital sphere [49].

The results also indicate important protective factors. Most children and adolescents reported using active coping strategies, such as cognitive restructuring, seeking family support, and problem-solving, and these were perceived as the most helpful strategies. Further, personal, familial, and social resources were strongly associated with better mental health outcomes. Children and adolescents with higher levels of these resources had substantially lower odds of low HRQoL, mental health problems, and internalizing symptoms. This highlights the potentially important role of supportive interpersonal environments and individual strengths in relation to better mental health outcomes during times of crises. Strengthening such resources – both within families and in educational and community settings – may therefore represent a relevant target for preventive and supportive interventions. This aligns with recent recommendations from the German National Academy of Sciences, which emphasises the central importance of fostering self-regulation competencies as key foundations for healthy development and resilience in the face of societal challenges [50]. This is particularly important for children and adolescents who are at elevated risk due to social disadvantage or parental mental illness. For these groups, targeted support in leisure, educational, and health-care settings is essential to ensure equitable opportunities for good mental health.

A major strength of this study is its population-based longitudinal design and the availability of data from eight measurement waves collected since the beginning of the COVID-19 pandemic. The use of validated instruments and harmonized survey procedures across all waves ensures comparability over time and allows robust insights into long-term trends.

This study has several limitations. Although repeated assessments allow the observation of temporal patterns, the design does not permit causal conclusions, and the reported associations – particularly regarding crisis-related future anxiety, social media use, and mental health outcomes – should not be interpreted causally. Methods explicitly accounting for repeated measurements, such as mixed-effects models or generalized estimating equations, would provide stronger inferential evidence regarding within-individual change. Given the number of repeated comparisons across waves and outcomes, the possibility of Type I error and false-positive findings cannot be fully excluded. In addition, the study relies on self-reports and parental proxy reports, which may be influenced by subjective perceptions and reporting biases. The composite risk group classification combines heterogeneous structural and familial risk factors and therefore does not allow for a differentiated interpretation of the relative contribution of individual risk factors. Likewise, the odds ratios reported in the risk and resource analyses should be interpreted cautiously, as they may be influenced by common-method variance, dichotomization, overlap in construct content, and model specification choices. As with all online surveys, selective participation and selective attrition across waves cannot be fully excluded, particularly among more vulnerable families. Although sample replenishment and weighting procedures were used to maintain representativeness, and sociodemographic and socioeconomic indicators remained stable across waves, weighting primarily improves demographic representativeness and cannot fully correct for attrition bias over time. Finally, the German-only administration may have contributed to an underrepresentation of families with migration backgrounds, which may affect generalizability and could lead to an underestimation of the vulnerable risk group and associated psychosocial burden in the population. In addition, the BELLA reference period (2014–2017) represents a pre-pandemic benchmark but not an immediately pre-COVID baseline, and broader secular changes in adolescent mental health and digital environments should therefore also be considered when interpreting differences over time.

Taken together, the findings of this eighth COPSY wave emphasize that youth mental health in Germany remains under notable strain for a significant subgroup of young people in the context of overlapping global crises. Although gradual improvements since the pandemic peak are encouraging, the persistence of elevated distress compared to pre-pandemic levels as reflected by the BELLA reference period (2014–2017) indicates an ongoing need for public health attention. At the same time, the majority of young people report levels of well-being within the normal range, underscoring considerable resilience in the youth population. The strong association between future anxiety and mental health, and the protective role of coping resources should be important considerations for prevention, intervention, and policy. Strengthening familial and social support systems, promoting adaptive coping, and improving access to mental health services – especially for high-risk groups – may represent relevant targets for supportive and preventive interventions. Continued monitoring of youth mental health remains essential to identify trends early and to inform targeted, evidence-based responses that support both vulnerable subgroups and the broader youth population.

As this manuscript provides an overview of the main descriptive findings of the eighth COPSY wave, future research should further examine the temporal and causal relationships between global crises, future anxiety, social media use, coping, and mental health outcomes. In particular, the development and empirical testing of a conceptual model of youth mental health in times of multiple crises may help to better understand how macro-level stressors interact and affect psychological well-being over time.

## Supplementary Information

Below is the link to the electronic supplementary material.

Supplementary Material 1

## Data Availability

The data that support the findings of this study are available from the corresponding author upon reasonable request.
