# Supplementary material for: Current global crises and youth mental health in Germany: the role of media use, coping and resources
Source: Eur Child Adolesc Psychiatry. 2026 Jun 13;35(10):3213–26. doi: 10.1007/s00787-026-03092-6 (PMC13696573; doi:10.1007/s00787-026-03092-6)
Supplement: Supplementary file 1 [file 787_2026_3092_MOESM1_ESM.docx]

**Supplementary Material**

**Current Global Crises and Youth Mental Health in Germany: The Role of Media Use, Coping and Resources**

European Child & Adolescent Psychiatry

Anne Kaman^1#^, Janine Devine¹, Michael Erhart¹^,2^, Ann-Kathrin Napp¹^,3^, Franziska Reiss¹^,3^, Christine M. Freitag^4^, Fionna Zoellner¹, Ulrike Ravens-Sieberer¹^,3#^

¹ Department of Child and Adolescent Psychiatry, Psychotherapy, and Psychosomatics, University Medical Center Hamburg-Eppendorf, Hamburg, Germany

^2^ Alice Salomon University of Applied Science, Berlin, Germany

^3^ German Center for Child and Adolescent Health (DZKJ), partner site Hamburg, Germany

^4^ Department of Child and Adolescent Psychiatry, Psychosomatic Medicine and Psychotherapy, University Hospital Frankfurt, Goethe University, Frankfurt am Main, Germany

**^#^Corresponding authors:** Dr. Anne Kaman (a.kaman@uke.de) & Prof. Dr. Ulrike Ravens-Sieberer (ravens-sieberer@uke.de)

Supplementary Figure S1. Participant flow across the eight waves of the COPSY study

Supplementary Table S1. Prevalence Estimates and 95% Confidence Intervals for HRQoL and Mental Health Outcomes Across COPSY Waves (T0–T8)

| Wave | Low HRQoL | | | Mental health problems | | | Anxiety symptoms | | | Depressive symptoms | | | Loneliness | | |
| --- | --- | --- | --- | --- | --- | --- | --- | --- | --- | --- | --- | --- | --- | --- | --- |
|  | % | CI low | CI high | % | CI low | CI high | % | CI low | CI high | % | CI low | CI high | % | CI low | CI high |
| T0 | 15.30 | 13.01 | 17.58 | 17.61 | 15.72 | 19.51 | 17.49 | 15.07 | 19.90 | 15.01 | 12.74 | 17.27 | 14.28 | 12.06 | 16.50 |
| T1 | 40.21 | 37.20 | 43.22 | 30.41 | 28.14 | 32.67 | 24.11 | 21.49 | 26.74 | 17.99 | 15.63 | 20.35 | 34.60 | 31.68 | 37.52 |
| T2 | 47.75 | 44.76 | 50.74 | 30.96 | 28.71 | 33.21 | 30.06 | 27.31 | 32.80 | 24.30 | 21.73 | 26.87 | 38.55 | 35.63 | 41.46 |
| T3 | 35.12 | 32.39 | 37.85 | 27.01 | 24.84 | 29.17 | 26.79 | 24.26 | 29.32 | 19.66 | 17.38 | 21.93 | 27.02 | 24.48 | 29.56 |
| T4 | 41.02 | 38.14 | 43.90 | 28.46 | 26.30 | 30.63 | 27.83 | 25.20 | 30.45 | 21.28 | 18.88 | 23.67 | 29.82 | 27.14 | 32.50 |
| T5 | 27.33 | 24.73 | 29.93 | 22.61 | 20.62 | 24.60 | 24.78 | 22.26 | 27.30 | 14.20 | 12.17 | 16.24 | 22.29 | 19.86 | 24.71 |
| T6 | 22.32 | 19.95 | 24.68 | 23.19 | 21.17 | 25.21 | 21.30 | 18.98 | 23.63 | 10.67 | 8.92 | 12.42 | 17.20 | 15.06 | 19.34 |
| T7 | 20.77 | 18.31 | 23.24 | 22.14 | 20.04 | 24.24 | 22.99 | 20.43 | 25.54 | 13.08 | 11.03 | 15.13 | 20.54 | 18.08 | 22.99 |
| T8 | 22.44 | 19.96 | 24.91 | 22.42 | 20.38 | 24.46 | 25.01 | 22.44 | 27.58 | 15.23 | 13.10 | 17.36 | 18.28 | 15.98 | 20.57 |

Supplementary Table S2. Full logistic regression models for risk and resource factors associated with mental health outcomes

|  | **Health-related quality of life**  **(KIDCREEN)** | | **Mental health problems**  **(SDQ)** | | **Depressive**  **Symptoms**  **(CES-DC)** | | **Anxiety**  **Symptoms**  **(SCARED)** | |
| --- | --- | --- | --- | --- | --- | --- | --- | --- |
|  | **OR** | **95% CI** | **OR** | **95% CI** | **OR** | **95% CI** | **OR** | **95% CI** |
| Belonging to risk group | 1.49 | 1.01-2.20 | 2.02 | 1.31-3.12 | 1.22 | 0.86-1.75 | 1.41 | 0.93-2.14 |
| Pronounced resources | 0.06 | 0.04-0.10 | 0.11 | 0.07-0.19 | 0.20 | 0.15-0.28 | 0.14 | 0.09-0.22 |
| Gender (Ref. Male) |  |  |  |  |  |  |  |  |
| Female | 0.88 | 0.47-1.68 | 0.90 | 0.44-1.83 | 2.29 | 1.39-4.06 | 2.34 | 1.11-4.93 |
| Age groups (Ref. 11-13 years) |  |  |  |  |  |  |  |  |
| 14-17 years | 1.04 | 0.61-1.79 | 1.35 | 0.75-2.41 | 1.45 | 0.87-2.49 | 2.06 | 1.05-4.04 |
| 18-21 years | 1.12 | 0.62-2.01 | 0.55 | 0.27-1.15 | 1.16 | 0.65-2.08 | 2.43 | 1.20-4.95 |
| Gender * Age group |  |  |  |  |  |  |  |  |
| Female * 14-17 years | 1.28 | 0.57-2.90 | 0.47 | 0.18-1.20 | 0.67 | 0.32-1.38 | 0.54 | 0.22-1.34 |
| Female * 18-21 years | 1.16 | 0.50-2.71 | 0.89 | 0.31-2.56 | 0.80 | 0.37-1.73 | 0.33 | 0.13-0.86 |
| Hosmer-Lemeshow GoF | *p*=0.011 | | *p*=0.425 | | *p*<0.095 | | *p*=0.334 | |
| Nagelkerke R-square | 0.317 | | 0.210 | | 0.168 | | 0.176 | |
